# Thermal Annealing Induced Controllable Porosity and Photoactive Performance of 2D ZnO Sheets

**DOI:** 10.3390/nano10071352

**Published:** 2020-07-11

**Authors:** Yuan-Chang Liang, Chen-Shiang Hung, Wei-Cheng Zhao

**Affiliations:** Department of Optoelectronics and Materials Technology, National Taiwan Ocean University, Keelung 20224, Taiwan; willia2121@gmail.com (C.-S.H.); qaz5263153@gmail.com (W.-C.Z.)

**Keywords:** ZnO, thermal annealing, porosity, crystalline quality, photoactive performance

## Abstract

Porous ZnO sheets containing various degrees of a nanoscaled pore were successfully synthesized using a simple hydrothermal method and various postannealing procedures. The porosity features of the ZnO sheets can be easily tuned by changing both the annealing temperature and annealing atmosphere. The dense porous nature of ZnO sheets is beneficial to enhance light absorption. Moreover, the substantially increased oxygen vacancies in the ZnO sheets were observed especially after the hydrogen treatment as revealed in the X-ray photoelectron spectroscope and photoluminescence analyses. The high density of surface crystal defect enhanced the photoinduced electron-hole separation rate of the ZnO sheets, which is crucial for an improved photoactivity. The porous ZnO sheets formed at a hydrogen atmosphere exhibited superior photoactive performance than the porous ZnO sheets formed at the high-temperature ambient air annealing. The dense pores and massive crystal defects formed by a hydrogen atmosphere annealing in the ZnO crystals might account for the observed photoactive behaviors in this study.

## 1. Introduction

Two dimensional (2D) sheet- or plate-like metal oxides for scientific applications attract much attention recently due to their unique physical and chemical properties [1,2,3,4]. The 2D sheet- or plate-like oxide nanomaterials are beneficial in increasing the light interaction area in the oxides and are promising in photoactive device applications. Porous oxide nanomaterials made of these 2D oxides are reported to exhibit excellent photocatalytic and photoactivated properties [5,6,7]. The porous ZnO sheets being ultrathin, mesoporous, and single-crystalline have been shown as advantageous in photocatalytic degradation of methylene blue [5]. The excellent solar-driven photocatalytic hydrogen evolution has been observed for the mesoporous black anatase TiO_2_ nanosheets synthesized via a biotemplate method combined with an ethanediamine encircling process [6]. Chemical bath derived porous 2D β-Bi_2_O_3_ nanoplates show excellent photoinduced current density and have potential in the application of photoelectron catalysis [7]. For photocatalytic applications, both faces of 2D sheets and pores can be in contact with the target pollutant solution, hence favoring the sufficient utilization of the catalytic surface. The aforementioned examples demonstrate that the 2D oxide nanostructures fabricated with a suitable pore number and size in them can further successfully enhance the photoactive performance of these 2D oxide nanomaterials.

ZnO is an attractive wide band gap semiconductor oxide. It possesses a high chemical stability, electrochemical coupling coefficient, and impressive photostability [8,9]. Development of 2D ZnO nanostructures via various routes is therefore of potential interest for photoactive device applications. Liang et al. have synthesized Al-doped ZnO nanosheets via electrodeposition [10]. A soft solution chemical bath deposition method has been adopted to prepare 2D polar-surface-dominated ZnO nanosheets [11]. Moreover, the porous 2D ZnO structures have also been reported. For example, a hierarchically assembled porous rectangular ZnO plate has been synthesized through the calcination of a hydrozincite (Zn_5_(CO_3_)_2_(OH)_6_) intermediate [12]. The previous work fabricates the porous ZnO nanosheets from a one-step polyol refluxing method [13]. However, the synthesis of porous 2D ZnO structures via a one-step synthesis route or only by calcination of the hydrozincite intermediate is not promising to modulate the pore size and density in the parent structure.

Hydrothermal crystal growth of ZnO has been widely developed recently to design the morphology of ZnO crystals at a low cost and processes temperature. The development of 2D ZnO nanosheets synthesis processes via a hydrothermal crystal growth triggers more attention because such a synthesis route has flexible process parameters and is promising to modulate the corresponding shape dependent properties of ZnO. However, further controlling the pore formation with varied size and distribution density is still a challenge. Moreover, the previous works on porous ZnO sheets do not well demonstrate the charge separation issues based on the systematical photoelectrochemical works [14,15,16,17]. Notably, in addition to the pore size of the ZnO sheets on photocatalytic degradation towards organic dyes as investigated by previous works, the reports on the effect of hydrogen annealing engendered crystal defects in the porous ZnO sheets on photoactive performance are limited in number. The difference from the above previous porous ZnO sheet-related works is that the current research focuses on the photoactive performance of various porous ZnO sheets with a controllable pore size and crystal defect density variations; moreover, the photoinduced charge separation capability of various ZnO sheet-like samples are correlated with the rigorous photoelectrochemical works. The experimental results herein provide an importance reference to design 2D porous ZnO nanostructures with desirable photoactive performance and the proposed process development approach is promising to integrate porous ZnO sheets in various photoactivated nanodevice applications. The structure-dependent photoactive performances of various porous ZnO sheets are systematically investigated in this study to understand the feasibility of applying this porous ZnO sheet to high-efficiency photo-activated devices.

## 2. Materials and Methods

For preparation of ZnO sheets, 2.23 g of zinc nitrate hexahydrate (Zn(NO_3_)_2_·6H_2_O), together with 8.34 g of urea ((NH_2_)_2_CO), was dissolved in 100 mL of deionized water. The resulting solution was stirred by using a magnetic stirrer for 1 h, and 20 mL of the precursor solution was transferred into a Teflon-lined autoclave. The F-doped tin oxide (FTO) glass slides were used as substrates for the deposition of ZnO sheets. All cleaned substrate were perpendicularly suspended in a solution, and hydrothermal treatments were carried out at 90 °C for 4 h. The substrates were taken out of autoclave at room temperature and washed with ethanol and deionized water for several times and dried in air at 60 °C. The formation mechanism of ZnO sheets via the precursor solution adding urea and conducted with the adequate thermal annealing procedure is shown below: Urea can be used as a stabilizer during hydrothermal ZnO crystal growth. When urea is dissolved in an aqueous precursor solution, it will slowly be displaced by water molecules to produce ammonia and carbonate anion (CO_3_^2−^). During the hydrothermal process, urea decomposed and produced NH_3_. NH_4_^+^ ions will generate from NH_3_ ions and markedly increase pH aqueous solution and support the hydrothermal ZnO crystal growth process ((1) and (2) reactions) [18]:

CON_2_H_4_ + 3H_2_O → CO_2_ (g) + 2NH_3_H_2_O (1)

2NH_3_ · H_2_O + CO_2_ → 2NH_4_^+^ + CO_3_^2−^ + OH^−^ (2)

For the ZnO precursor aqueous, zinc nitrate hexahydrate will provide Zn^2+^ ions when dissolved in water as in

Zn(NO_3_)_2_ · 6H_2_O + H_2_O → Zn^2+^ + 2NO_3_^−^ + 7H_2_O (3)

4Zn^2+^ + CO_3_^2−^ + 6OH^−^ + H_2_O → Zn_4_CO_3_(OH)_6_ · H_2_O(s) (4)

Zn_4_CO_3_(OH)_6_ · H_2_O → 4ZnO (s) + 4H_2_O + CO_2_ (g) (5)

Zn_4_CO_3_(OH)_6_·ZnO sheet-like crystals are formed during the hydrothermal reactions in an aqueous precursor solution [19], and they are further decomposed to crystalline ZnO sheet-like crystals after annealing at 300 °C in ambient air. Some of the crystalline ZnO sheets are respectively further conducted in thermal annealing procedures at 500 °C in ambient air and at 450 °C in hydrogen atmosphere to form a porous sheet structure. The notations for the as-synthesized porous ZnO sheets formed at high-temperature ambient air and hydrogen atmosphere annealings are respectively ZnO-A and ZnO-H in this study.

The sample crystal structure was evaluated by X-ray diffraction (XRD; Bruker D2 PHASER) analysis using Cu Kα radiation. The sample morphology and detailed microstructures were characterized by scanning electron microscopy (SEM) and transmission electron microscopy (TEM) with a Hitachi S-4800 (Tokyo, Japan) and a Philips Tecnai F20 G2 (Amsterdam, The Netherlands), respectively. The optical properties were investigated by recording the (UV–vis) diffuse reflectance spectra at room temperature using a Jasco V750 spectrophotometer in the ultraviolet to visible light wavelength range. X-ray photoelectron spectroscopy (XPS) was carried out with an ULVAC-PHI PHI 5000 VersaProbe spectrometer (Chigasaki, Japan) using Mg Kα radiation for sample elemental analysis. Photoluminescence (PL) spectra were measured on a luminescence spectrophotometer (HORIBA HR800, Kyoto, Japan) with an excitation wavelength of 325 nm at room temperature. The photoelectrochemical experiments were performed using a three-electrode electrochemical working station, where the as-synthesized sheet-like sample was used as the working electrode, the Pt wire was used as the counter electrode. An Ag/AgCl reference electrode in an aqueous solution containing 0.5 M Na_2_SO_4_ was employed during the measurements. The Nyquist plots of various samples were measured at the open circuit potential with the frequency from 1 Hz to 100 kHz using an electrochemical impedance spectroscopy (EIS, SP150, Seyssinet-Pariset, France). Photodegradation tests of various sheet-like samples (approximately 10 mg sheets are used for test) were performed by comparing the degradation of 10^−5^ M aqueous solution of rhodamine B (RhB) under full-spectrum irradiation excited from the 100 W Xe arc lamp at various irradiation durations.

## 3. Results and Discussion

**Figure 1:**
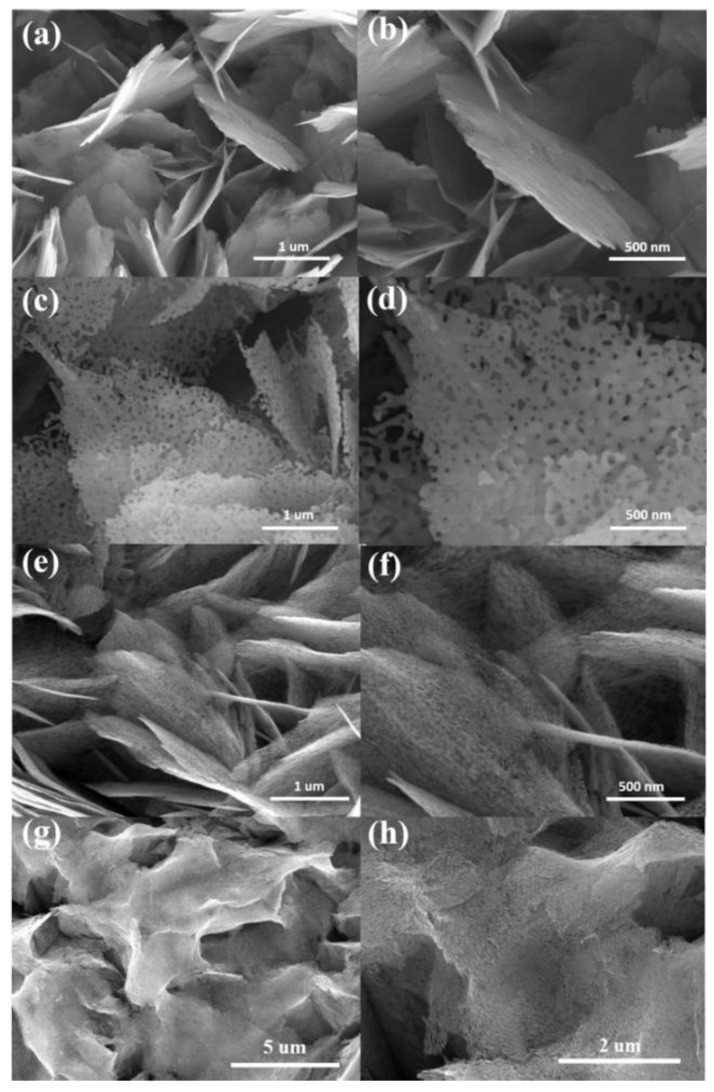
Top-view SEM images of various ZnO sheets at different magnifications: (**a**,**b**) ZnO. (**c**,**d**) ZnO-A. (**e**,**f**) ZnO-H. (**g**,**h**) The ZnO sheets conducted with a hydrogen annealing procedure at a temperature above 500 °C.

Figure 1a,b shows the typical morphology of as-synthesized ZnO sheets. The ZnO sheets have an uneven surface feature. This might be associated with the crystalline ZnO sheet comes from the 300 °C postannealed as-grown hydrothermally derived two-dimensional sheet-like Zn_4_CO_3_ (OH)_6_·H_2_O. Such a thermal annealing procedure might engender the surface contractile change of the Zn_4_CO_3_ (OH)_6_·H_2_O, and therefore a rugged surface feature was formed on the crystalline ZnO sheets. The ZnO sheets showed a sheet layer thickness of less than 20 nm, and a few microns in length and width. No aggregation was observed on the ZnO sheet-like sample; all the ZnO sheets were freestanding and exhibited a good dispersibility over the area of the interest. After the high temperature annealing procedure (500 °C) in ambient air and the hydrogen atmosphere (450 °C), the ZnO-A (Figure 1c,d) and ZnO-H (Figure 1e,f) sheets show the nanometer-sized porous architectures were formed in the sheets. The ZnO sheet-like products well maintained the morphology without significant collapse after the annealing procedures, but with slightly decreased thickness. Notably, the ZnO sheets conducted with a hydrogen annealing procedure at a temperature above 500 °C induced seriously sheet appearance curling and reunion in this work (Figure 1g,h), showing a more intense morphology transformation of the solid ZnO sheet structure to porous ZnO sheet structure at a higher hydrogen annealing temperature. Therefore, the 450 °C hydrogen annealing temperature is the optimal temperature to obtain the ZnO sheets with a desirable porous structure. Compared with the sheet morphology of the ZnO-A in Figure 1c,d, the ZnO-H sheets in Figure 1e,f exhibited smaller and denser pores in the sheets. Considering their high porosity, these as-synthesized porous ZnO sheet structures are highly favorable to be applied in light absorption and a high surface area.

**Figure 2:**
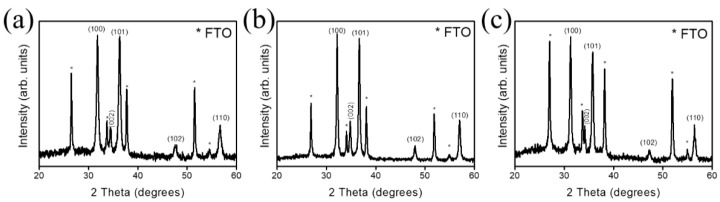
XRD patterns of various ZnO sheets: (**a**) ZnO. (**b**) ZnO-A. (**c**) ZnO-H. The asterisk denoted the Bragg reflections from the F-doped tin oxide (FTO).

Figure 2a–c shows the XRD patterns of the ZnO, ZnO-A, and ZnO-H sheets. The XRD patterns display several distinct and sharp Bragg reflections of hexagonal wurtzite ZnO (100), (002), (101), (102), and (110) (JCPDS no. 00-036-1451). The (100) and (101) preferred orientation dominated the crystallographic feature of ZnO sheets herein. No characteristic peaks for any other impurity phase were observed. A similar non-polar plane dominated crystallographic feature has also been reported in the electrochemical derived ZnO nanosheets [10]. Notably, the angle position of the Bragg reflections for the ZnO-H sample is slightly shifted to a lower angle position. For examples, the Bragg reflection of (100) of the ZnO, ZnO-A, and ZnO-H sheets appeared at approximately 31.78, 31.80, and 31.42°, respectively. The calculated interplanar distance values for ZnO, ZnO-A, and ZnO-H are 0.281, 0.281, and 0.285 nm, respectively. The interplanar distance of the (101) for the ZnO, ZnO-A, and ZnO-H sheets is respectively 0.248, 0.247, and 0.250 nm. The slightly larger lattice volume was observed in the ZnO sheets conducted with the hydrogen atmosphere annealing procedure. The shift of Bragg reflections of ZnO nanowires prepared by the carbothermal reduction process towards a lower angle has also been shown after the hydrogen atmosphere annealing procedure [20]. This could be caused by the reasons that hydrogen is incorporated into the ZnO lattice as an interstitial, and possibly introducing oxygen defects will result in expansion of the lattice [20,21].

**Figure 3:**
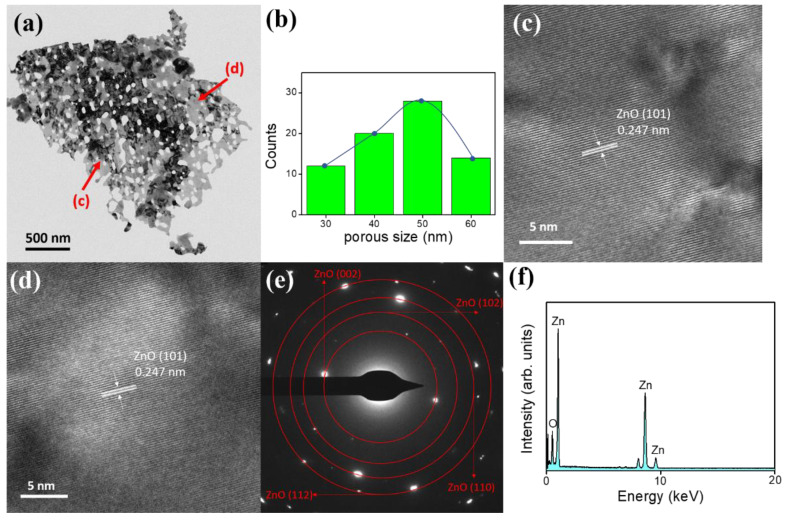
TEM analysis of the ZnO-A sheet: (**a**) low-magnification image. (**b**) Histogram of pore size. (**c**,**d**) HRTEM images. (**e**) SAED pattern. (**f**) EDS spectrum.

Figure 3a exhibits the typical TEM image of a ZnO-A sheet, from which it can be clearly seen that the individual sheet had the width of approximately 2 um. The sheet was full of irregular pores of tens of nanometers and these pores are randomly distributed in the sheets. The corresponding histogram of the calculated porous size distribution of the ZnO-A sheet is shown in Figure 3b and the average pore size of the ZnO-A sheet is approximately 48 nm in this study. The high-resolution (HR) TEM images taken from the local regions in Figure 3a are displayed in Figure 3c,d. The orderly and clear lattice fringes, parallel to each other, show that the ZnO sheet are well-crystallized, and the resolved spacing between two neighboring parallel fringes is approximately 0.247 nm, which is in good agreement with the aforementioned XRD pattern with the (101) plane of hexagonal wurtzite ZnO. The diffraction spots in the corresponding selected area electron diffraction (SAED) pattern (Figure 3e) were identified as the (002), (102), (110), and (112) reflections of hexagonally structured ZnO, indicating that the ZnO sheet was in a good crystallinity. Energy dispersive X-ray spectroscopy (EDS) shows that the ZnO-A sheet (Figure 3f) is elementally composed of zinc and oxygen. The O/Zn atomic ratio was evaluated to be 0.97. The ZnO-A sheet with a highly elemental purity was formed herein.

**Figure 4:**
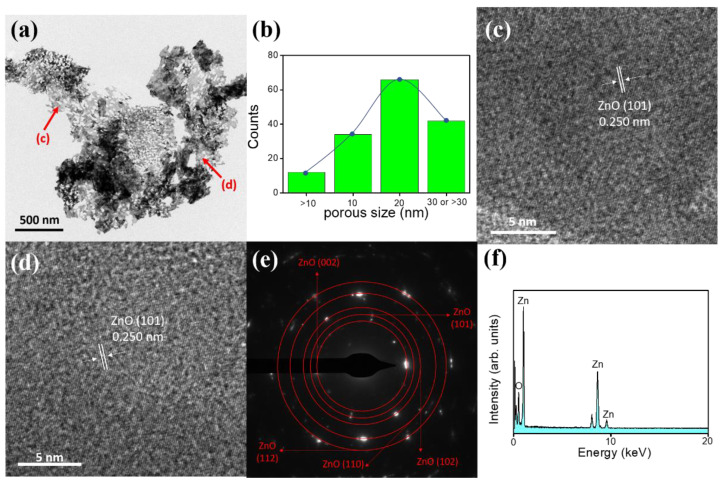
TEM analysis of the ZnO-H sheet: (**a**) low-magnification image. (**b**) Histogram of pore size. (**c**,**d**) HRTEM images. (**e**) SAED pattern. (**f**) EDS spectrum.

Figure 4a exhibits a low-magnification TEM image of the ZnO-H sheet, from which it can be clearly seen that the individual sheet was full of nanosized pores with the diameter of a few tens of nanometers. The pore size distribution was more homogeneous and pore density was higher herein in comparison with those of the ZnO-A sheet as exhibited in Figure 3a. The corresponding histogram of the calculated pore size distribution of the ZnO-H sheet is shown in Figure 4b. The average pore size of the ZnO-H was approximately 21 nm, which was significantly smaller than that of the ZnO-A sample. The pore size analysis herein was consistent with the SEM observations. The HRTEM images taken from various regions of the ZnO-H are displayed in Figure 4c,d. In the HR images, the lattice fringes showed the imaging characteristics of the hexagonal wurtzite ZnO crystal (JCPDS no. 00-036-1451), in which the interplanar spacing of 0.250 nm corresponded to the distance of the ZnO (101) crystallographic plane. Notably, the HRTEM images here exhibited a relatively poor crystalline quality than did the ZnO-A, which was due to the introduction of more oxygen defects into the ZnO lattices and engendered lattice expansion and reduced crystallinity, consistent with the earlier XRD peak shift result obtained. The SAED pattern of this sheet is displayed in Figure 4e. The pattern exhibited (002), (101), (102), (110), and (112) reflection rings of wurtzite-type ZnO, and this indicates that the sheet was polycrystalline. Figure 4f is an EDS spectrum taken from this ZnO-H sheet, which revealed the presence of Zn and O in the sheet structure. The evaluated O/Zn atomic ratio was approximately 0.92 herein. A slightly higher degree of oxygen deficiency of the ZnO-H sheet was observed. The above results confirmed that the porous ZnO sheet with a homogeneous pore distribution was successfully grown on the FTO substrate via the hydrothermal method combined with the subsequent annealing process in a hydrogen atmosphere.

**Figure 5:**
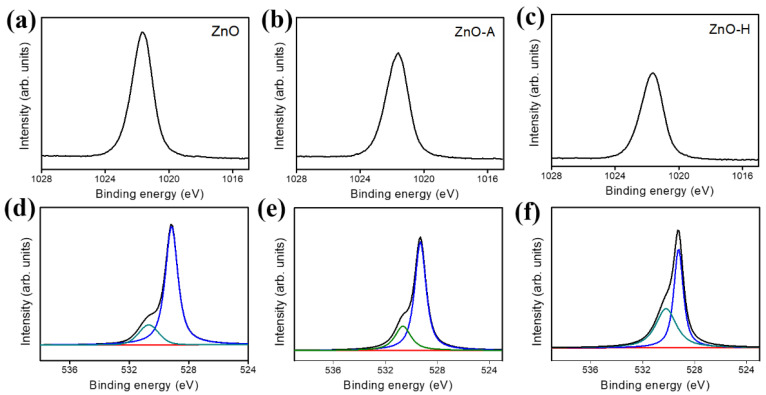
Zn 2p narrow scan spectra of various ZnO sheets: (**a**) ZnO. (**b**) ZnO-A. (**c**) ZnO-H. O1s narrow scan spectra of various ZnO sheets: (**d**) ZnO. (**e**) ZnO-A. (**f**) ZnO-H.

The formation of as-synthesized ZnO sheets were obtained after postannealing Zn_4_CO_3_(OH)_6_·H_2_O crystals at 300 °C. The XPS survey scan spectrum (Figure S1) of as-synthesized ZnO sheets also demonstrated no nitrogen existed in the sample, revealing the well phase transformation of Zn_4_CO_3_(OH)_6_·H_2_O to ZnO after the postannealing procedure. Figure 5a–c displays Zn2p_3/2_ spectra of various ZnO sheets. The binding energies of Zn2p_3/2_ for various ZnO sheets are around 1021.7 eV, revealing that Zn^2+^ ions existed in the ZnO lattice [22,23,24]. Figure 5d–f show the O1s XPS spectra of various ZnO sheets. For all ZnO sheets, the O1s peaks centered around 530.6 eV, and the peak profiles were asymmetric, a visible shoulder peak appeared at a higher binding energy. The O1s core-level spectra herein suggested the possible presence of at least two different kinds of oxygen binding states in these ZnO sheets. The deconvolution of the O1s spectra demonstrated that the subpeak centered at 529.7 eV could be indexed to the lattice oxygen of ZnO, and the subpeak at the higher binding energy of 530.6 eV was attributed to a contribution of oxygen ions that were in oxygen-deficient regions within the ZnO lattice and chemisorbed species on the sample surface [24]. Surface oxygen vacancies can induce localized states close to the conduction band of ZnO and act as a donor, which might affect the electronic properties of the oxides. The XPS O1s analysis results revealed that the oxygen vacancy density of the ZnO-H sheets was higher compared to ZnO and ZnO-A sheets.

**Figure 6:**
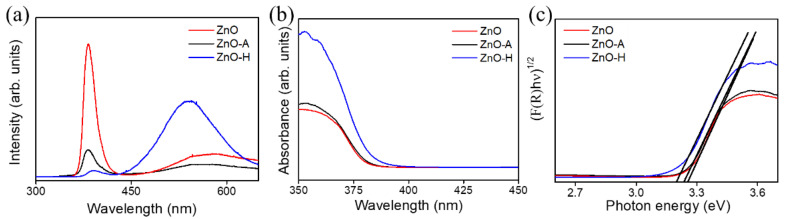
(**a**) The photoluminescence (PL) spectra of various ZnO sheets. (**b**) UV–vis diffuse reflectance spectra of various ZnO sheets. (**c**) Bandgap energies of various ZnO sheets.

Figure 6a shows the PL spectra of the ZnO, ZnO-A, and ZnO-H sheets, which provided information about the crystal defects presented in the ZnO crystal. The ZnO sheets had a narrow and sharp emission peak at the UV region. The UV emission peak is the intrinsic bandgap absorption of ZnO, which comes from the recombination of free excitons [25]. Compared with ZnO-A and ZnO-H sheets, the ZnO sheets had a substantially intense UV emission band. It shows that the pristine ZnO sheets had a higher electron-hole recombination rate. The substantially quenched UV emission band intensity for the ZnO-A and ZnO-H, demonstrated superior charge separation ability in these porous sheets. Notably, the UV emission band of the ZnO and ZnO-A sheets was located at approximately 382 nm, and the UV emission band was red-shifted to a higher wavelength of 387 nm for the ZnO-A sheets. The ZnO sheets annealed in a hydrogen atmosphere might incur a high density of crystal defect in the ZnO lattice, and lead to lattice expansion. This has been associated with the slight red shift in the near band edge emission of the ZnO crystal in the PL analysis [26]. Furthermore, a distinct visible light emission band appeared in the ZnO, ZnO-A, and ZnO-H sheets. These broad emission bands are associated with the charged oxygen vacancy, which originated from the surface electron traps associated with ionized oxygen vacancies from the defect donor level to the valence band [27]. Comparatively, the visible light emission band intensity of the ZnO-H sheets was well above that of the ZnO and ZnO-A sheets. Hence, the PL emission spectra results showed that the ZnO sheets annealed in a hydrogen atmosphere owned more surface oxygen vacancies. Moreover, the substantial removal of surface absorbed OH group at suitable annealing conditions has been shown in an obviously blue shift in the visible region peak of the ZnO crystals [28]; this might account for the observed blue shift in the visible region peak of the ZnO-H sheets in comparison with that of the ZnO and ZnO-A sheets herein. Figure 6b shows the UV–vis diffuse reflectance spectra for the ZnO, ZnO-A, and ZnO-H sheets. The diffuse reflectance measurements were converted into the equivalent absorption coefficient using the Kubelka–Munk (K–M) method: F(R) = (1 − R)^2^/2R = α, in which R is the reflectance and α is the absorption coefficient [29]. The light absorption edges of various ZnO sheets were located at approximately 370–390 nm. The absorbance spectra showed that the ZnO-H sheets exhibited a red-shift, which occurred in the absorption edge and presented a significantly enhanced absorption in the UV and visible-light region. The increase of visible light absorption for the ZnO-H sheets can be attributed to the introduction of crystal defects in the band-gap energy levels of ZnO during annealing in a hydrogen atmosphere [30]. However, in comparison with the pristine ZnO sheets, the ZnO-A sheets only showed a slightly enhanced optical absorption ability. The suitable amount of pores between and within the ZnO sheets is posited to be advantageous for enhanced light absorption due to the multi-reflection of trapped incident light within the nanostructures [31]. In this way, the optical path length for light transport through the ZnO-H sheets because of its high pore density and small pore size is longer, resulting in a greater absorption capacity herein. Such an enhanced absorption capacity is beneficial for photoactive applications. Figure 6c depicts the direct bandgap energies of various ZnO sheets estimated from a plot of (F(R)*hν*)^2^ vs. photo energy (*hν*) according to the K–M model in which *h* is Planck’s constant, and *ν* is the frequency of light. [29]. The estimated bandgap energies are respectively 3.25, 3.24, and 3.20 eV for the ZnO, ZnO-A, and ZnO-H sheets. The Urbach energy was further used to evaluate the defect density size of various ZnO sheets. We have plotted ln(F(R)) against the photon energy (Figure S2) according to the proposed equation [32]. The reciprocal of the slope of the linear fit, below the optical band gap region, gives the value of Urbach energy of the ZnO, ZnO-A, and ZnO-H sheets and that value was approximately 71, 76, and 110 meV, respectively. It can be seen that the Urbach energy of the ZnO sheets markedly increased after annealing at a hydrogen atmosphere, which might be attributed to an increased number of oxygen defect center in the system [33].

**Figure 7:**
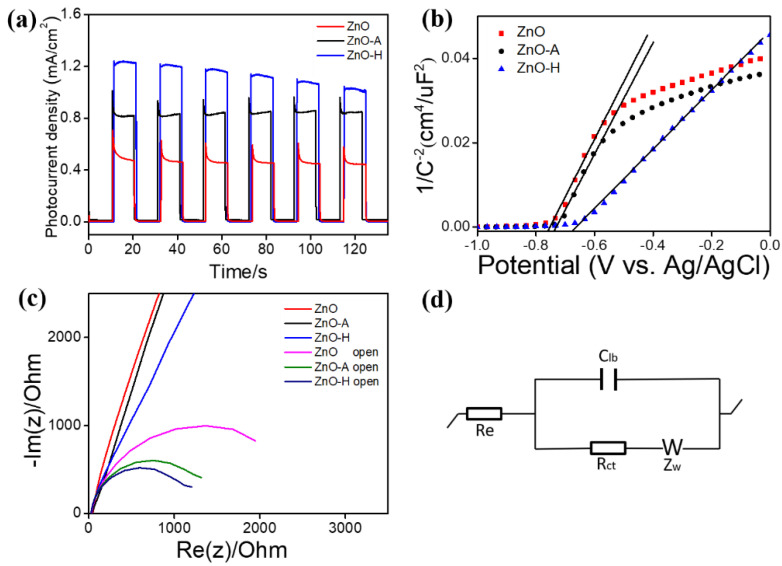
(**a**) Photocurrent density versus time curves of various ZnO sheets with chopped light irradiation. (**b**) Mott–Schottky plots. (**c**) Nyquist plots. (**d**) A possible equivalent circuit used to evaluate Rct value of various ZnO sheets.

Figure 7a shows the photoresponse performances of the ZnO, ZnO-A, and ZnO-H sheets under 0.5 V (vs. Ag/AgCl potential). A fast photocurrent response was observed for each switch on/off event in various sheet photoelectrodes. However, when the light was turned on, the ZnO and ZnO-A photoanodes all showed a distinct spike-like transient response. The possible cause of this spike transient feature was associated with the whole process of the back-reaction, which is regarded as the recombination of the photoinduced electrons and holes at the surface states of the oxides [34]. In addition, the larger initial anode spike of the ZnO sheets were substantially decreased after a hydrogen treatment, indicating that the electron-hole recombination was more effectively suppressed on the ZnO-H sheets. Moreover, the steady-state photocurrent density of the ZnO sheets was enhanced with a porous structure and a hydrogen treatment. The average photocurrent intensities of the ZnO, ZnO-A, and ZnO-H sheets were approximately 0.49, 0.84, and 1.23 mA/cm^2^ under illumination. The ZnO-H showed the highest photocurrent density at the test cycles. The results herein revealed that the dense porous nature of ZnO sheets is beneficial to enhance light absorption, and the increased crystal defects after the hydrogen treatment increase the photoinduced electron-hole separation time, which is crucial for improved photoactivity. This was supported by the earlier UV–vis and PL analyses. The improvement of the photocurrent of the annealed ZnO sample verified that the charge separation efficiency improved greatly through the ambient air and hydrogen annealing treatments. However, the photocurrent density of the ZnO-H sheets declined from 1.23 to 1.02 mA/cm^2^ after six cycles. This can be attributed to the excessively small porous on the surface of the ZnO nanosheet after the hydrogen treatment, which could not withstand photocorrosion during the photoelectrochemical (PEC) experiments. This may cause such a decline in durability of the photoactivity [35]. Figure 7b shows the Mott–Schottky (M–S) plots of the ZnO, ZnO-A, and ZnO-H sheets, which were generated based on capacitances that were derived from the EIS obtained at each potential with 1 kHz frequency in the dark. As expected for n-type nature, all the ZnO sheets exhibited positive slopes in the M–S plots. The carrier density of the oxide semiconductor was inversely proportional to the slope of the straight-line portion in the M–S plot according to the proposed relationship of 1/C^2^ versus the applied potential in the literature [36]. The slope of the straight-line portion in the M–S plot for the ZnO-H sheets was the lowest among various sheet-like samples, revealing an increased carrier density. This could be associated with the formation of surface oxygen vacancies of hydrogen-annealed ZnO sheets. Hydrogen treatment at an elevated temperature will bring about desorption of partial oxygen on the ZnO surface and made the ZnO surface positively charged. This might cause new doping energy levels formed under the ZnO conduction band [37]. The annealing-induced formation of doping energy levels with a small difference in energy level with respect to the ZnO conduction band is feasible for the electrons in the doping energy levels to easily transfer to the ZnO conduction band and thus enhances the carrier density of ZnO [38]. Based on the aforementioned, the ZnO-H sheets showed a relatively smaller slope compared to the ZnO and ZnO-A sheets as expected in this work. The higher carrier density in the oxide semiconductors reduced their bulk resistance and was beneficial for improving their photoactive performance. A similar M–S curve slope variation feature between the porous ZnO and pristine ZnO structures has been reported in the chemical solution derived ZnO crystals [39]. The comparison of interface charge separation efficiency for various ZnO sheets was further investigated by the Nyquist plots with and without irradiation in Figure 7c. The radius of the arc on the Nyquist plots reflects the interface layer resistance occurring at the surface of electrode. The smaller arc radius implies higher efficiency of the charge transfer ability [40,41]. The results in Figure 7c showed that the decreasing order of the semicircular radius of the ZnO sheets with formation of a porous structure by conducting thermal annealing procedures. Figure 7d shows the possible equivalent circuit for evaluating the charge-transfer resistance (R_ct_) of the various ZnO sheet-based electrodes. In Figure 7d, R_e_ is the series resistance, C_lb_ is the equivalent electrical circuit component, and Z_w_ is the Warburg impedance [42]. According to the fitting results, the R_ct_ values of the ZnO, ZnO-A, and ZnO-H sheets in the dark were respectively 12,902, 12,021, and 10,131 ohm, while the R_ct_ values of the ZnO, ZnO-A, and ZnO-H under irradiation were approximately 1877, 1087, and 914 ohm, respectively. It illustrated that the porous structure is an aid to the improvement of charge transfer efficiency and the electron transfer rate on the ZnO sheet-based electrode. Overall, the EIS results behaved that the ZnO-H sheets exhibited superior charge separation and transportation efficiencies, in turn reduced the interfacial resistance.

**Figure 8:**
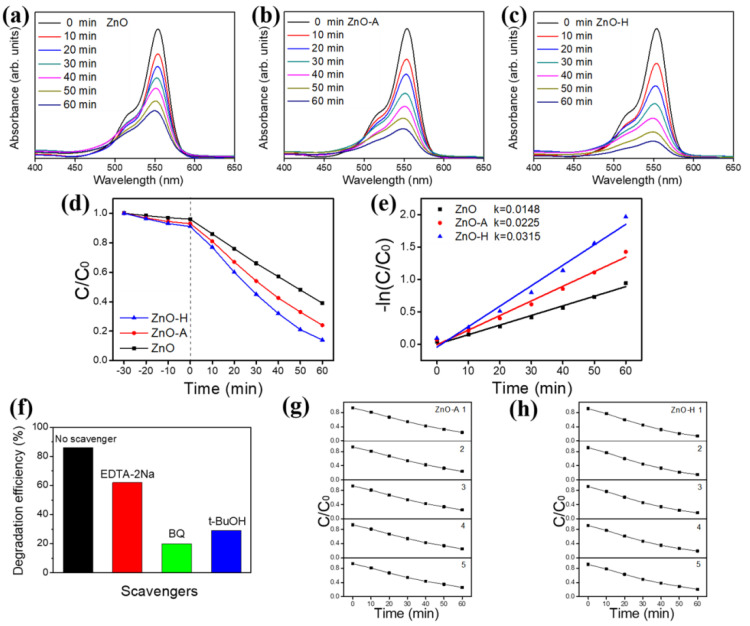
Absorption spectra intensity variation of the RhB dye solution as a function of irradiation time with various ZnO sheets as photocatalysts: (**a**) ZnO. (**b**) ZnO-A. (**c**) ZnO-H. (**d**) C/Co vs. irradiation time plot. (**e**) The reaction rate constants of various ZnO sheets. (**f**) Scavenger tests of RhB solution containing ZnO-H photocatalyst. (**g**) The cyclic photodegradation of RhB solution with the ZnO-A. (**h**) The cyclic photodegradation of RhB solution with the ZnO-H.

Figure 8a–c shows the irradiation duration dependent characteristic absorbance spectra intensity variation of aqueous RhB solution using various ZnO sheets as photocatalysts. The intensity of the absorbance spectra decreased with irradiation duration, suggesting that the RhB dyes were gradually photodegraded by various ZnO sheets. Comparatively, the drop degree of the absorbance spectra intensity is more intense for the RhB solution with the ZnO-H sheets at the given irradiation condition. For comparison, the photodegradation abilities of various ZnO sheets are displayed in Figure 8d. The C/C_o_ = I_t_/I_o_ was used to evaluate the photodegradation degree, in which C_o_ and C are the initial and residual concentration of the RhB dye at t = 0 and at any irradiation duration t, respectively and I_t_ and I_o_ are respectively the characteristic absorbance spectra intensities at irradiation duration t and at t = 0 [40,43]. Both porous ZnO sheets, i.e., ZnO-A and ZnO-H photocatalysts exhibited higher photocatalytic activities than pristine ZnO sheets. Nearly complete photodegradation (approximately 86%) of the RhB dyes was achieved for the RhB solution containing ZnO-H after 60 min irradiation; ZnO-A photodegraded 76% RhB dyes under the same irradiation duration. By contrast, the pristine ZnO sheet exhibited a relatively low photodegradation performance and only degraded 61% RhB dyes after 60 min irradiation. Notably, the dark adsorption balance tests of various ZnO sheets were also conducted to understand the dye adsorption capability of various ZnO sheets. The C/C_o_ decreased 4% for the pristine ZnO sheets under the 30 min dark balance condition; the C/C_o_ decreased to 7% and 9% under the 30 min dark balance condition. The results herein demonstrated that the ZnO-H sheets displayed a higher degree of dye adsorption. The hydrogen annealing ZnO sheets visibly exhibited superior photodegradation degree towards the RhB solution among various ZnO sheet photocatalysts after deducting the contribution of dye adsorption on the sample surface. The kinetic linear simulation curves of the photocatalytic RhB degradation for different sheet-like photocatalysts are exhibited in Figure 8e; an apparent first-order kinetic model for photodegradation reactions at low initial concentrations was observed. The kinetic model follows the formula −ln(C/C_o_) = kt, where k is the first-order rate constant (min^−1^) and t is irradiation duration [38]. Figure 8e shows the −ln(C/C_o_) versus irradiation time curves of various sheet-like photocatalysts on photodegrading RhB dyes. The k was found to be 0.0148 min^−1^, 0.0225 min^−1^, and 0.0315 min^−1^ for the ZnO, ZnO-A, and ZnO-H photocatalysts, respectively. The photodegradation performance of various sheet-like photocatalysts follows the order: ZnO-H > ZnO-A > ZnO. Obviously, the tiny pores in ZnO sheets impact their photocatalytic performance, which may be due to the increased light absorption and larger surface area for dye adsorption. It has been shown that the porous structure was beneficial to provide more photocatalytic reaction centers for the absorption of reactant organic molecules. A similar porous structure effect has been demonstrated in mesoporous TiO_2_ materials, in which an increased number of active sites in a mesoporous structure effectively increases their photocatalytic activity [44]. Furthermore, a porous structure is also effective for light adsorption and thus generates more photoinduced electrons and holes [45]. The as-prepared ZnO-H sheets can work as effective photocatalysts herein. The corresponding photocatalytic reaction process can be formulated as following [46]:

ZnO + hv → e^−^ + h^+^ (6)

e^−^ + O_2_ → ⋅O_2_^−^ (7)

h^+^ + OH^−^ → ·OH (8)

·O_2_^−^, h^+^, ·OH + RhB → oxidation products (9)

When ZnO sheets are irradiated by light with energy higher or equal to the band gap, an electron (e^−^) in the valence band (VB) can be excited to the conduction band (CB) with the simultaneous generation of a hole (h^+^) in the VB. Excited state e^−^ and h^+^ can recombine and get trapped in metastable surface states, or react with electron donors and electron acceptors adsorbed on the semiconductor surface. The photoelectron is easily trapped by electron acceptors like adsorbed O_2_ to form ·O_2_^−^ radical, whereas the photoinduced hole can be easily trapped by an electronic donor, such as OH^−^ or organic pollutants, to further oxidize organic dyes [47]. Notably, the photoactive ability of the ZnO sheets does not only depend on the surface adsorption ability, but also relates to the concentration of oxygen defects on the surfaces. Oxygen vacancies on ZnO sheet surface can serve as the electron capturing center to restrain the recombination of e^−^ and h^+^ [48]. An increased oxygen vacancy number of the ZnO sheets annealed in a hydrogen atmosphere effectively increased their photocatalytic efficiency. The active species were also investigated to discuss the possible photocatalytic mechanism. The h^+^, ∙O_2_^−^, and ∙OH are the probable active species taking part in RhB dyes photodegradation. In this study, the edentate disodium (EDTA-2Na), benzoquinone (BQ), and tert-Butyl alcohol (t-BuOH) were used as the traps for h^+^, ∙O_2_^−^, and ∙OH in the photodegradation reaction, respectively. The photocatalytic activity of ZnO-H decreased slightly by the addition of EDTA-2Na and decreased largely with the addition of BQ or t-BuOH, indicating that the ∙O_2_^−^ and ∙OH are the main active species in the RhB dye photodegradation process (Figure 8f). The photostability of the porous ZnO sheets (ZnO-A and ZnO-H) was further investigated by cyclic photodegradation experiments in Figure 8g,h. The photodegradation rates of the ZnO-A and ZnO-H decreased respectively by approximately 2% and 5% after five consecutive cycles. The ZnO-A photocatalyst maintained excellent photoactive performance after cyclic photodegradation tests; however, the ZnO-H photocatalyst demonstrated fair photoactive performance after cyclic photodegradation tests, indicating that the retention of photodurability of the ZnO-H sheets still remains a challenge. The summarized comparative photodegradation performances of RhB solution containing various ZnO or porous ZnO sheets from literatures are shown in Table S1 [5,49,50,51]. Nevertheless, the porous ZnO-H sheets herein demonstrated desirable photocatalytic degradation capability towards the RhB solution.

## 4. Conclusions

In summary, porous ZnO sheets were successfully synthesized using a methodology consisting of a simple hydrothermal method and post-annealing procedures. The porosity features of the ZnO sheets can be easily tuned by changing the annealing temperature and annealing atmosphere. The experimental results demonstrated that, compared with the pristine ZnO sheet, the porous ZnO sheets demonstrated enhanced optical absorption ability. The suitable amount of pores between and within the ZnO sheets is posited to be advantageous for enhanced light absorption due to multi-reflection of trapped incident light within the porous nanostructures. Moreover, the porous structure in the ZnO sheets impacted their photocatalytic performance, which may be due to the increased light absorption, larger surface area for dye adsorption, and increased photoexcited charge separation efficiency. The experimental results herein demonstrate the strategy of thermal annealing induced porosity in the ZnO sheet-like structure is promising to design the 2D ZnO crystals with various degrees of photoactive performance for photoactivated device applications.

## Supplementary Materials

The following are available online at https://www.mdpi.com/2079-4991/10/7/1352/s1, Figure S1: The XPS survey scan spectrum of as-synthesized ZnO sheets, Figure S2: Plot of Urbach energy for ZnO, ZnO-A, ZnO-H sheets, Table S1: The summarized comparative photodegradation performances of RhB solution containing various ZnO or porous ZnO sheets from literatures.
