# Supplementary material for: Thermal Annealing Induced Controllable Porosity and Photoactive Performance of 2D ZnO Sheets
Source: Nanomaterials (Basel). 2020 Jul 11;10(7):1352. doi: 10.3390/nano10071352 (PMC7407819; doi:10.3390/nano10071352)
Supplement: Supplementary file 1 [file nanomaterials-10-01352-s001.pdf]

Thermal Annealing Induced Controllable Porosity and Photoactive Performance of 2D ZnO Sheets

Table S1. The summarized comparative photodegradation performances of RhB solution containing various ZnO or porous ZnO sheets from literatures [5,49-51]:

Catalyst	Synthesis method	Dye concentration	Catalyst amount	Time (min)	Degradation (%)	Light power
ZnO-H porous sheets (this work)	Hydrothermal	20 ml, 10^{-5} M	10 mg	60	86	100 W
ZnO mesoporous nanosheets	Hydrothermal	100 ml, 10^{-5} M	100 mg	150	94	158 W
Flower-like ZnO nanosheets	Hydrothermal	50 ml, 10^{-6} M	20 mg	30	99	300 W
ZnO porous nanosheets	Hydrothermal	60 ml, $7x10^{-6}$ M	30 mg	60	88	250 W
ZnO nanosheets	Antisolvent process	20 ml, 10^{-5} M	10 mg	120	92	N/A

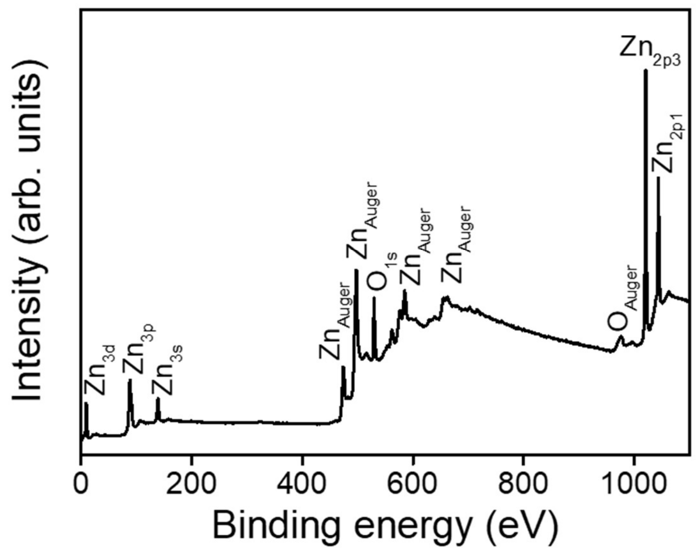

Figure S1. The XPS survey scan spectrum of as-synthesized ZnO sheets.

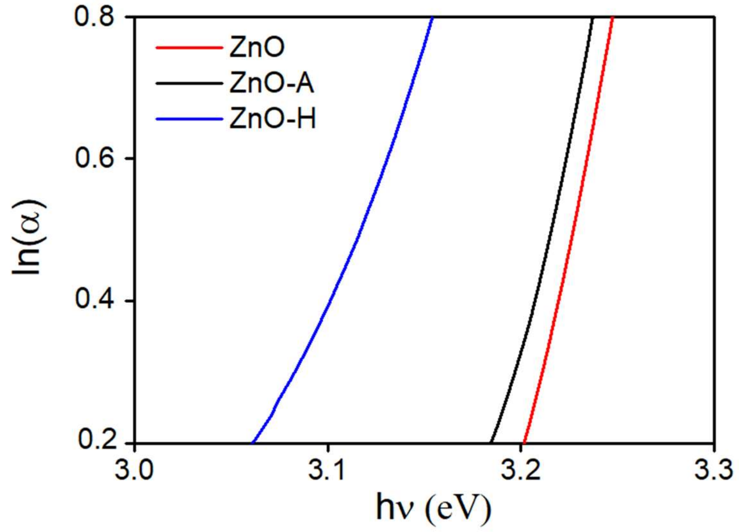

Figure S2. Plot of Urbach energy for ZnO, ZnO-A, ZnO-H sheets.
